# The whole-genome sequence and analysis of *Alternaria alternata* strain LG038 isolated from *Capsicum annuum*

**DOI:** 10.1128/mra.01195-25

**Published:** 2026-02-04

**Authors:** Chao Ma, Kelei Han, Dankan Yan

**Affiliations:** 1 Institute of Plant Protection and Agro-Products Safety, Anhui Academy of Agricultural Sciences 667051 , Hefei, Anhui, China; Rochester Institute of Technology, Rochester, New York, USA

**Keywords:** *Alternaria alternata*, genome analysis, the whole-genome sequence

## Abstract

The genome of *Alternaria alternata* strain LG038, isolated from pepper fruits, was sequenced using a combination of Illumina paired-end and PacBio HiFi technologies. The final assembly has a size of 34,546,397 bp and a GC content of 50.74%.

## ANNOUNCEMENT

*Alternaria* is a genus of fungi that is widely distributed worldwide and can be found in various natural substrates, functioning as saprophytes, parasites, and endophytes. Complete genome sequences of *A. alternata* isolates have been obtained from plants such as *Actinidia chinensis*, *Arachis hypogaea*, and *Sclerocarya birrea* with genome sizes ranging from 32.0 to 33.8 Mb (1–3). However, whole-genome sequencing of *A. alternata* on peppers remains scarce. Whole-genome sequencing serves as a crucial tool for comprehensively understanding the biology and characteristics of *A. alternata*. It is crucial for elucidating pathogenic mechanisms and developing effective control strategies against *A. alternata*.

The fungal isolate was designated as strain LG038, isolated from diseased pepper fruits (*Capsicum annuum* variety “Jiusheng”) in Hexian County, Anhui Province, China (118°37′4″, 31°18′9″), collected in March 2024. Tissue fragments (approximately 5 mm × 5 mm) were excised from the lesion margins at the junction of diseased and healthy tissues. Surface disinfection was performed using 75% ethanol, followed by rinsing with sterile water, and then it was inoculated onto potato dextrose agar (PDA) plates (4). After single spore selection and purification, strain LG038 was cultured for 7 days, and then the mycelia were scraped from the PDA plate for total genomic DNA extraction using the OMEGA Fungal DNA Extraction Kit. Based on the *TEF*, ITS, *GPD*, and *Alat1* sequences, specific amplification primers were designed (Table 1). The amplification of the target sequences and subsequent Sanger sequencing, followed by BLAST search on the NCBI website, identified the strain as *A. alternata*. DNA quality was assessed using a Nanodrop 2500 (Thermo Scientific, USA). The genomic DNA was sent to Majorbio Bio-pharm Biotechnology Co., Ltd. (Shanghai, China) for sequencing.

**TABLE 1:** Primers for amplifying partial LG038 gene segments for Sanger sequencing

Name	Sequence (5′–3′)	Target genes
GDF	GCCGTCAACGACCCCTTCATTGA	*GPD*
GDR	GGGTGGAGTCGTACTTGAGCATGT	
ITS1	TCCGTAGGTGAACCTGCGG	ITS
ITS4	TCCTCCGCTTATTGATATGC	
TEF1-728F	CATCGAGAAGTTCGAGAAGG	*TEF*
TEF1-986R	TACTTGAAGGAACCCTTACC	
Alta1F	ATGCAGTTCACCACCATCGC	*Alta1*
Alta1R	ACGAGGGTGATGTAGGCGTC	

Libraries were constructed using the Illumina paired-end sequencing platforms (Illumina NovaSeq X Plus) and the PacBio HiFi (PacBio Sequel IIe) (5, 6). For Illumina sequencing, DNA was fragmented, and libraries were constructed with the NEXTFLEX Rapid DNA-Seq Kit, followed by PCR amplification and paired-end sequencing. NovaSeqTM X Plus Control Software (Version 1.3.1, default parameters) was used for Illumina data. The raw data were quality-controlled using fastp software (Version 0.20.0, default parameters) to obtain high-quality clean reads (7). The sequencing produced a total of 38,871,426 reads, with an initial Raw *Q*30 score of 94.82% and a read length of 151 bp. After quality filtering, 37,509,688 clean reads were retained, achieving a Clean *Q*30 of 95.26%.

Following quality control assessment by Illumina sequencing, PacBio sequencing involved fragmenting DNA, preparing SMRT Bell libraries, and detecting base incorporation signals in real time, with the PacBio platform-based third-generation sequencing employing built-in calibration, negating further quality measures. Base calling was conducted with SMRT Link (Version 25.3, default parameters) for PacBio HiFi data. The PacBio HiFi library yielded a total of 133,386 reads, comprising 1,367,013,228 bp.

The genome was *de novo* assembled using hifiasm (Version 0.19.5, default parameters) with the quality-controlled reads from the PacBio HiFi data, while Illumina sequencing data were utilized solely for quality control assessment (8). Assembly quality was assessed using BUSCO (Version 5.4.5, fungal database parameters) and CEGMA (Version 2.5, default parameters) (9, 10). The final assembly consisted of 35 scaffolds (>1,000 bp), with a total length of 34,546,397 bp (34.5Mb). The scaffold *N*50 value is 3,324,522 bp and the BUSCO completeness is 98.7%. The coverage of the genome is 39.57×. The assembled genome sequence has been deposited in the NCBI database. The longest scaffold measured 8,319,787 bp, while the GC content was 50.74%. Using tRNAscan-SE (Version 2.0.12, -E for eukaryotic tRNA search, -L for mitochondrial tRNA search) and Barrnap (Version 0.9, -kingdom euk to specify the eukaryotic kingdom, -lencutoff 0.8, *E*-value 1e−06) analysis, a total of 195 tRNAs and 72 rRNAs were identified in the genome (11, 12).

Gene prediction of LG038 was performed using MAKER 2 (Version 2.32, default parameters), resulting in 11,915 putative protein-coding genes with a total length of 21,780,467 bp (13). The average gene density was 0.34/kb, and the length of intergenic regions was 12,765,930 bp. The Database of Fungal Virulence Factors (DFVF) was used to identify virulence-related genes. Predicted protein sequences were aligned against DFVF using Diamond (Version 0.8.35, *E*-value 1e−5), yielding 1,172 virulence-related genes in the sequenced strain LG038. Furthermore, using Diamond (Version 0.8.35, *E*-value 1e−5) to align predicted protein sequences against the Comprehensive Antibiotic Resistance Database (Version 1.1.3), 31 antibiotic resistance-related genes were identified in LG038.

## Data Availability

The partial gene sequences of strain LG038, including the translation elongation factor (TEF), internal transcribed spacer (ITS), glyceraldehyde-3-phosphate dehydrogenase (GPD), and Alternaria major allergen (Alta1), have been deposited in the GenBank database under accession numbers PV405411, PV104005.1, PV405407, and PV405409. The assembled genome sequence is available under the GenBank assembly accession number JBRAXB000000000.1, BioProject accession number PRJNA1328952, and BioSample number SAMN51363658. The raw data have been deposited in the NCBI Sequence Read Archive (SRA) under temporary submission ID SRR35749014 and BioProject accession number PRJNA1338339.
